# Potential Impact of Next-Generation Weight Loss Drugs on Cancer Incidence

**DOI:** 10.1001/jamanetworkopen.2025.30904

**Published:** 2025-09-08

**Authors:** Darren R. Brenner, Yibing Ruan, Chantelle Carbonell

**Affiliations:** 1 Department of Oncology, Cumming School of Medicine, University of Calgary, Calgary, Alberta, Canada; 2 Department of Community Health Sciences, Cumming School of Medicine, University of Calgary, Calgary, Alberta, Canada; 3 Department of Cancer Epidemiology and Prevention Research, Cancer Care Alberta, Calgary, Alberta, Canada

## Abstract

This decision analytical model projects changes in obesity-related cancer incidence among US adults following the potential impact of glucagon-like peptide 1 receptor agonists on weight loss.

## Introduction

Excess body size has emerged as an impactful component of population-level cancer risk. Current data suggest that at least 13 cancer sites are convincingly associated with excess body size, most often characterized by a body mass index (BMI; calculated as weight in kilograms divided by height in meters squared) greater than 25.0. These associations have been confirmed by multiple large observational studies, pooled in meta-analyses, and reviewed by international panels.^1^

Excess body size has been steadily rising for the past 40 years in Western populations, including the United States, partly due to the consumption of ultra-processed, energy-dense, nutrient-poor foods and the influence of built environments and daily work-life routines.^2^ Recent trial data suggest next-generation weight loss drugs (NGWLDs), including glucagon-like peptide-1 receptor agonists (GLP-1RAs), may help combat obesity-related cancers (ORCs). GLP-1RAs have shown great promise in weight reduction among patients with type 2 diabetes and individuals at high cardiometabolic risk.^3^ These results have been impressive, especially when compared with supervised diet and activity modification programs, which have shown minimal long-term impact in the placebo arms of these trials. Analyses of administrative datasets have also provided early evidence for a potential preventive role of GLP-1RAs in reducing the risk of ORCs.^4^

## Methods

This population-based decision analytical model estimated the potential impact of GLP-1RAs on reducing the incidence of ORCs in the United States. We extracted cancer incidence data from GLOBOCAN from 2025 through 2050.^5^ Relative risks (RRs) for BMI and cancer were extracted from the American Institute for Cancer Research Continuous Update Project.^1^ We modeled a 1-year phase-in for the full effect of weight loss in 2025 and a 5-year latency period between weight loss and its effects on cancer incidence. We modeled BMI distribution by sex based on National Health and Nutrition Examination Survey data from 2021 to 2023 and assumed a fixed BMI distribution after 2023. We estimated the potential impact fractions (PIFs) and 95% CIs of preventable ORCs, based on the confidence interval of the RR, from a 10% reduction in body weight for individuals with BMI 30.0 or greater and with an 80% probability of weight reduction for those with BMI between 27.0 and 30.0, based on GLP-1RA trial data. Additional modeling details are included in the eAppendix in Supplement 1.

This study used publicly available, deidentified data and did not involve human participants directly; thus per the Tri-Council Policy Statement, review and approval by an ethics committee were not required. Data analysis was conducted from December 2024 to February 2025. All analyses we conducted in R version 4.4.1 (R Project for Statistical Computing), and statistical significance was set at a 2-sided *P* < .05.

## Results

Under current overweight and obesity prevalence (36.2% and 39.5% among male individuals and 28.8% and 41.4% among female individuals, respectively), we projected that, in 2030, 18.0% of all ORCs among female individuals and 14.3% among male individuals would be attributable to high BMI in the United States. Cancer incidence across the 13 associated sites was projected to increase by 26.6% between 2025 and 2050, with the largest relative increases observed among cancers with the strongest associations with obesity.

Our analyses suggest that a GLP-1RA–related 10% weight reduction could lead to a total reduction of 1 222 584 ORC cases among male and female individuals combined by 2050 (Figure). The largest prevention potential was projected for breast and endometrial cancers among female individuals and for kidney and liver cancers among both sexes. Site-specific preventable cases are presented in the Table.

**Figure.**
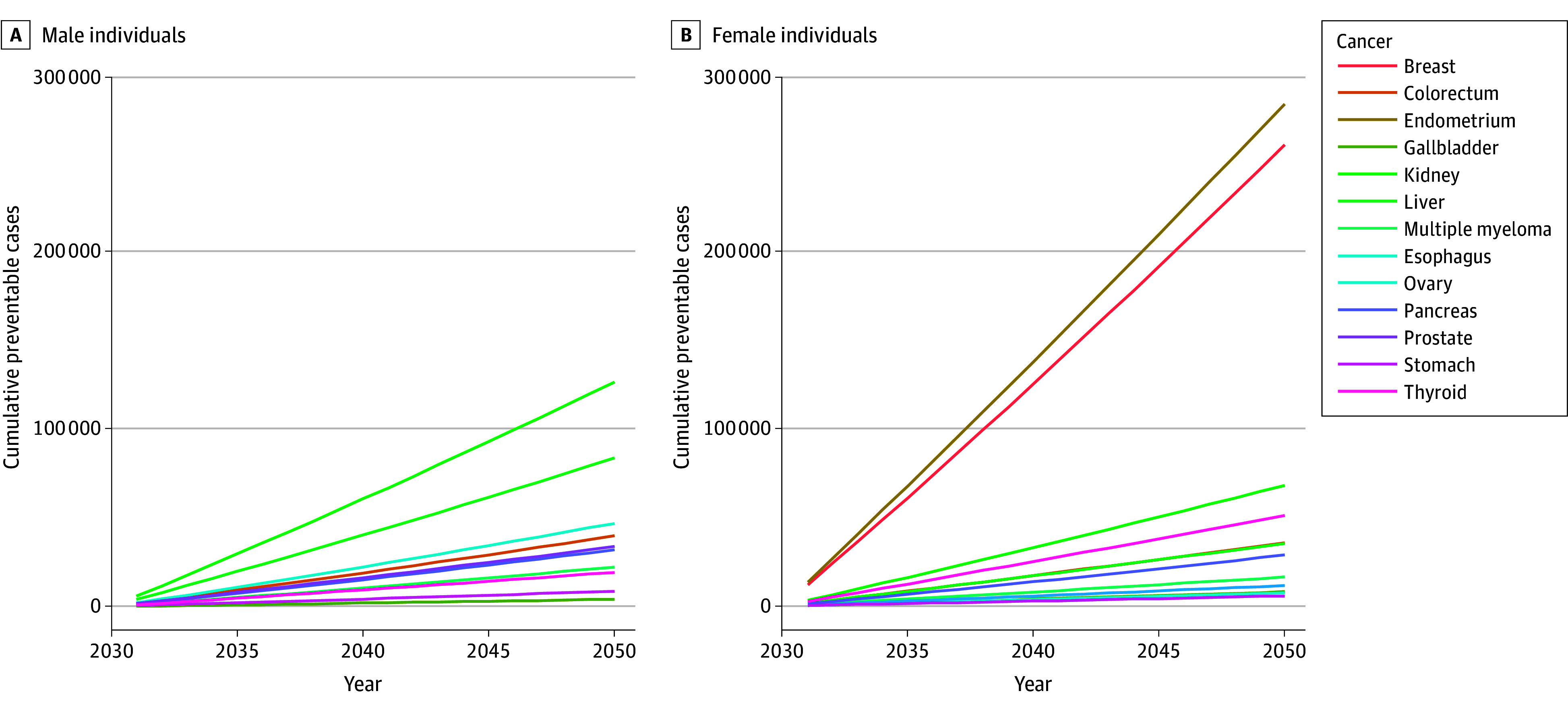
Site-Specific Projections of Preventable Cancers Associated With a 10% Reduction in Body Weight Among US Adults With Overweight and Obesity, 2031 to 2050

**Table.** Sex-Specific Estimates of Obesity-Related Cancers and PIFs of Preventable Cases With a 10% Reduction in Body Weight From the Use of Next-Generation Weight Loss Drugs, United States, 2031-2050

Cancer	Projected incidence, No.		2030, No. (95% CI)		2050, No. (95% CI)		Cumulative preventable cases, 2031-2050, No. (95% CI)
	2030	2050	PAF	Attributable cases	PIF from weight loss	Preventable cases	
**Female individuals**							
All obesity-related cancers	574 837	669 070	18.0 (16.0-19.9)	103 643 (92 062-114 107)	6.4 (5.7-7.1)	42 895 (37 821-47 571)	811 459 (715 549-900 778)
Breast	252 640	293 865	13.4 (10.4-16.6)	33 905 (26 168-41 813)	4.7 (3.6-5.9)	13 849 (10 619-17 315)	260 516 (199 747-325 724)
Colorectum	86 398	104 014	5.7 (3.3-7.8)	4882 (2881-6777)	1.8 (1.1-2.6)	1919 (1120-2684)	35 766 (20 873-50 032)
Endometrium	73 122	81 443	47.4 (41.0-54.2)	34 646 (29 987-39 635)	18.3 (15.3-21.3)	14 866 (12 454-17 352)	283 624 (237 595-331 044)
Gallbladder	3715	4571	26.3 (15.9-36.1)	978 (591-1341)	9.3 (5.3-13.3)	426 (242-607)	7894 (4481-11 248)
Kidney	27 969	32 304	31.0 (26.2-35.3)	8662 (7332-9876)	11.2 (9.3-12.9)	3605 (2991-4161)	68 066 (56 476-78 562)
Liver	14 259	16 938	31.0 (16.6-44.0)	4416 (2370-6281)	11.2 (5.7-16.8)	1890 (972-2844)	35 353 (18 173-53 184)
Multiple myeloma	16 322	19 493	13.2 (7.1-19.1)	2163 (1159-3120)	4.5 (2.3-6.5)	868 (456-1275)	16 260 (8540-23 874)
Esophagus	1848	2251	45.9 (35.8-55.4)	848 (662-1025)	17.6 (13.1-22.0)	396 (294-495)	7336 (5452-9179)
Ovary	23 494	27 469	6.8 (1.9-11.6)	1588 (457-2736)	2.2 (0.6-3.9)	609 (173-1063)	11 460 (3251-20 005)
Pancreas	33 615	41 903	11.1 (7.1-15.0)	3737 (2380-5040)	3.7 (2.3-5.1)	1553 (959-2119)	28 662 (17 688-39 105)
Stomach	2910	3502	24.4 (9.2-40.9)	710 (268-1189)	8.6 (3.0-15.2)	300 (105-532)	5600 (1966-9924)
Thyroid	38 545	41 317	18.4 (11.3-25.5)	7108 (4352-9846)	6.3 (3.8-8.9)	2614 (1559-3697)	50 922 (30 355-72 009)
**Male individuals**							
All obesity-related cancers	327 800	397 398	14.3 (12.4-16.1)	46 848 (40 745-52 892)	5.6 (4.9-6.4)	22 443 (19 416-25 497)	414 711 (358 835-471 186)
Colorectum	95 819	117 427	4.9 (2.9-6.9)	4703 (2785-6600)	1.8 (1.1-2.6)	2152 (1258-3005)	39 569 (23 132-55 270)
Gallbladder	1884	2442	22.8 (13.6-31.5)	429 (257-594)	9.1 (5.2-12.9)	222 (128-315)	4015 (2311-5703)
Kidney	52 352	62 484	26.8 (22.7-30.6)	14 044 (11 893-16 045)	10.9 (9.1-12.5)	6790 (5698-7823)	125 984 (105 719-145 144)
Liver	35 113	41 398	26.8 (14.5-38.5)	9420 (5091-13 535)	10.9 (5.6-16.4)	4499 (2303-6783)	83 426 (42 709-125 800)
Multiple myeloma	21 669	27 532	11.5 (6.2-16.6)	2487 (1354-3597)	4.4 (2.3-6.4)	1208 (636-1760)	21 982 (11 563-32 020)
Esophagus	12 062	14 867	40.1 (30.9-49.0)	4832 (3730-5911)	17.1 (12.7-21.7)	2542 (1892-3230)	46 656 (34 729-59 295)
Pancreas	37 512	47 890	9.6 (6.2-13.1)	3614 (2341-4926)	3.7 (2.3-5.0)	1753 (1122-2403)	31 820 (20 356-43 618)
Prostate	52 545	61 554	7.8 (4.0-11.7)	4080 (2127-6148)	2.9 (1.5-4.5)	1803 (926-2761)	33 570 (17 233-51 393)
Stomach	4496	5608	21.1 (7.9-34.9)	949 (355-1567)	8.4 (3.0-14.8)	469 (169-832)	8591 (3087-15 226)
Thyroid	14 348	16 195	16.0 (9.8-22.2)	2290 (1406-3186)	6.2 (3.7-8.8)	1005 (604-1432)	19 098 (11 482-27 223)

Abbreviations: PAF, population attributable fraction; PIF, potential impact fraction.

## Discussion

These results suggest that widespread use of GLP-1RAs among individuals with high BMI could considerably reduce ORC incidence in the US. These preliminary estimates are intended to stimulate discussion but should be interpreted with caution, as they are based on modeling data.

Our study is limited because we did not consider other measures of body fat—such as waist circumference, waist-to-hip ratio, or adult weight gain, all of which are risk factors distinct from BMI. While pharmacological intervention is neither ideal nor feasible for all individuals, the prevalence of chronic diseases and ORCs due to excess body size will continue to rise if left unaddressed. The potential impact of GLP-1RAs on cancer risk may be considerable given the number of individuals treated with these agents over the next few decades.

## Notes

Supplement 1. **eAppendix.** Technical Appendix
eReferences.

Supplement 2.
Data Sharing Statement
