# Supplementary material for: Potential Impact of Next-Generation Weight Loss Drugs on Cancer Incidence
Source: JAMA Netw Open. 2025 Sep 8;8(9):e2530904. doi: 10.1001/jamanetworkopen.2025.30904 (PMC12418124; doi:10.1001/jamanetworkopen.2025.30904)
Supplement: Supplement 1. — eAppendix. Technical Appendix eReferences. [file jamanetwopen-e2530904-s001.pdf]

Supplemental Online Content

Brenner DR, Ruan Y, Carbonell C. Next-generation weight loss drugs and cancer incidence. *JAMA Netw Open.* 2025;8(9):e2530904. doi:10.1001/jamanetworkopen.2025.30904

eAppendix. Technical Appendix

eReferences.

This supplemental material has been provided by the authors to give readers additional information about their work.

© 2025 Brenner DR et al. *JAMA Network Open*.

eAppendix. Technical Appendix

To estimate the preventable obesity-related cancer cases in USA, we applied the comparative risk assessment approach that has been extensively used to estimate the population attributable fractions (PAFs) due to risk factors and potential impact fractions (PIFs) from risk mitigations.[1] Quantitatively, we used body mass index (BMI) as a measure of body fatness and treated BMI as a continuous variable. For each sex-specific obesity-related cancer, PAF and PIF were calculated using the equation below.

$$PIF = \frac{\int^{R} R(x)P(x)dx - \int^{R} R(x)P^{*}(x)dx}{\int^{R} R(x)P(x)dx}$$

In this equation, x is the continuous variable of BMI from the lowest to highest value in US males or females. $RR(x)$ is a function of relative risk associated with an obesity-related cancer within the domain of x. $P(x)$ is the distribution of the exposure prevalence in the population. In this case, it is the distribution of BMI in US males or females. $P^{*}(x)$ is the counterfactual distribution of the exposure prevalence in the population with intervention. In this case, it is the BMI distribution after effective weight loss on population level. PAR is a special case of PIF in which the counterfactual distribution $P^{*}(x)$ of BMI is 100% under 25.0 kg/m^2. The preventable cases were estimated as the product of projected cancer cases and PIF.

The projected cancer incidence was acquired from IARC's Cancer Tomorrow Project, as part of the GLOBOCAN project.[2] Cancer Tomorrow predicts the future incidence and mortality of 36 cancer types in 185 countries or regions from the current estimates in 2022 up until 2050. We included 13 cancer types that have strong evidence to be causally linked to obesity, including oesophageal adenocarcinoma, cardia stomach, gallbladder, colorectum, liver, pancreas, kidney, thyroid, ovary, uterus, postmenopausal breast cancer, multiple myeloma and prostate cancer of advanced stages. For cancers with subtypes associated with obesity, we made the following assumptions: (1) the proportion of adenocarcinoma among all oesophageal cancers was 70% in males and 40% in females[3]; (2) 25% of all stomach cancers were cardia[4]; (3) average onset of menopause occurs at age 50 in females; (4) 20% of all prostate cancers are diagnosed at advanced stages.[5]

The relative risks (RR) associated with body mass index (BMI) were obtained from the Third Expert Report of the World Cancer Research Fund[6] or recent meta-analyses.[7,8] We assumed a loglinear relationship with RR for BMI between 25.0 to 50.0 kg/m^2.[9] When BMI is below 25.0 kg/m^2, we assumed an RR of 1.0. We also assumed a plateaued cancer risk beyond BMI of 50.0 kg/m^2 and no additional RR for BMI greater than 50.0 kg/m^2. We conducted sensitivity analyses by using the lower or upper confidence interval of the RR estimates for estimating PIF and preventable cases.

The distribution of BMI among US males and females was estimated from the NHANES database.[10] Information about anthropometric measurements including weight, height and BMI was obtained from examination data. The BMI distributions of males aged 20 and over, females aged 20 and over, and females aged 50 and over (for postmenopausal breast cancer) were estimated by combining the survey participants' recorded BMI with sampling weights. For the counterfactual distribution, we assumed that a 10% reduction in body weight would occur among those with BMI of 30.0 kg/m^2 and with an 80% probability among those with BMI between 27.0 and 30.0 kg/m^2, which

© 2025 Brenner DR et al. *JAMA Network Open*.

corresponds to a 10% decrease in BMI. For the main analysis, we used the data from the 2021-23 questionnaire and assumed the same BMI distribution in the future. We also assumed that the reduction in body weight occurs within a year and is sustained, and that there is a 5-year latency between weight reduction and cancer risk reduction. Therefore, an intervention that takes place in 2025 would show its full effect in 2031. We also conducted sensitivity analyses by using applying a 5% or 15% decrease in BMI.

As another sensitivity analysis, we projected an increasing trend in body fatness based on the NHANES examination data from 1999 to 2023. Average annual change in mean weight and height by sex was estimated with weighted linear regression. The weight and height distribution of the 2021-23 data was used as the base distribution in 2023. Starting from 2024, each survey participant was applied a random change in weight and height based on a normal distribution with the mean and standard error of the annual change estimated from the weighted linear regression. The BMI distribution was calculated with the changed weight and height and combined with the sampling weights. The counterfactual distribution as the main analysis (i.e., 10% reduction in body weight) was applied to the BMI distribution for 2026 onward. This process was repeated up to 2045, for which the intervention effect would be observed in 2050.

All statistical analyses were conducted using R Statistical Software (version 4.4).[11]

References

1. Shield KD, Parkin DM, Whiteman DC, et al. Population Attributable and Preventable Fractions: Cancer Risk Factor Surveillance, and Cancer Policy Projection. *Curr Epidemiol Rep*. Sep 2016;3(3):201-211. doi:10.1007/s40471-016-0085-5
2. Ferlay J, Laversanne M, Ervik M, et al. Global Cancer Observatory: Cancer Tomorrow (version 1.1). International Agency for Research on Cancer. Accessed 19 March 2025, https://gco.iarc.who.int/tomorrow/
3. Then EO, Lopez M, Saleem S, et al. Esophageal Cancer: An Updated Surveillance Epidemiology and End Results Database Analysis. *World J Oncol*. Apr 2020;11(2):55-64. doi:10.14740/wjon1254
4. Rustgi SD, McKinley M, McBay B, et al. Epidemiology of Gastric Malignancies 2000-2018 According to Histology: A Population-Based Analysis of Incidence and Temporal Trends. *Clin Gastroenterol Hepatol*. Dec 2023;21(13):3285-3295.e8. doi:10.1016/j.cgh.2023.01.037
5. Centers for Disease Control and Prevention. Prostate Cancer Incidence by Stage at Diagnosis. Updated 13 December 2024. Accessed 19 March 2025, https://www.cdc.gov/united-states-cancer-statistics/publications/prostate-cancer.html#cdc_research_or_data_summary_overview-introduction
6. World Cancer Research Fund/ American Institute for Cancer Research. *Diet, Nutrition, Physical Activity and Cancer: a Global Perspective 3rd edition*. 2018. *Continuous Update Project*. https://www.wcrf.org/dietandcancer/about
7. Kitahara CM, Platz EA, Freeman LEB, et al. Obesity and Thyroid Cancer Risk among U.S. Men and Women: A Pooled Analysis of Five Prospective Studies. *Cancer Epidemiology, Biomarkers & Prevention*. 2011;20(3):464-472. doi:10.1158/1055-9965.Epi-10-1220
8. Wallin A, Larsson SC. Body mass index and risk of multiple myeloma: a meta-analysis of prospective studies. *Eur J Cancer*. Jul 2011;47(11):1606-15. doi:10.1016/j.ejca.2011.01.020
9. Bhaskaran K, Douglas I, Forbes H, dos-Santos-Silva I, Leon DA, Smeeth L. Body-mass index and risk of 22 specific cancers: a population-based cohort study of 5·24 million UK adults. *Lancet*. Aug 30 2014;384(9945):755-65. doi:10.1016/s0140-6736(14)60892-8
10. Li M, Gong W, Wang S, Li Z. Trends in body mass index, overweight and obesity among adults in the USA, the NHANES from 2003 to 2018: a repeat cross-sectional survey. *BMJ Open*. Dec 16 2022;12(12):e065425. doi:10.1136/bmjopen-2022-065425
11. R Core Team. The R Project for Statistical Computing. Accessed 19 December 2024, https://www.r-project.org/

© 2025 Brenner DR et al. *JAMA Network Open*.
