# Supplementary material for: Potential Impact of Next-Generation Weight Loss Drugs on Cancer Incidence
Source: JAMA Netw Open. 2025 Sep 8;8(9):e2530904. doi: 10.1001/jamanetworkopen.2025.30904 (PMC12418124; doi:10.1001/jamanetworkopen.2025.30904)
Supplement: Supplement 2. — Data Sharing Statement [file jamanetwopen-e2530904-s002.pdf]

Data Sharing Statement

Brenner. Next-Generation Weight Loss Drugs and Cancer Incidence. *JAMA Netw Open*.
Published September 08, 2025. doi:10.1001/jamanetworkopen.2025.30904

Data
Data available: No

Additional Information
Explanation for why data not available: This study used publicly available data.
